# Continuity means “preserving a consistent whole”—A grounded theory study

**DOI:** 10.3402/qhw.v10.29872

**Published:** 2015-12-28

**Authors:** Malin Östman, Eva Jakobsson Ung, Kristin Falk

**Affiliations:** 1 Institute of Health and Care Sciences, Sahlgrenska Academy, University of Gothenburg, Gothenburg, Sweden; 2 Närhälsan Källstorp Health Centre, Trollhättan, Region Västra Götaland, Sweden; 3 Centre for Person-Centred Care (GPCC), Sahlgrenska Academy, University of Gothenburg, Gothenburg, Sweden

**Keywords:** Chronic disease, heart failure, life expectations, continuity, grounded theory method

## Abstract

Living with a chronic disease like chronic heart failure (CHF) results in disruptions, losses, and setbacks in the participants’ daily lives that affect health and well-being. By using grounded theory method, we illuminate whether persons with CHF experience discontinuity in life and, if so, what helps them to preserve and strengthen continuity in their daily lives. Thirteen individual interviews and one group interview with five participants, aged 62 to 88 years, were carried out. Through data collection and data analysis, we constructed three concepts that make up a model illustrating the participants’ experiences in daily life in relation to corporeality, temporality, and identity: *experiences of discontinuity*, *recapturing approaches*, and *reconciliation*. The first concept, *experiences of discontinuity*, was constructed from the following categories: *the alienated body*, *the disrupted time*, and *the threatened self*. The second concept, *recapturing approaches*, consists of categories with continuity creative constructions: *repossessing the body*, *maintaining a façade*, *seizing the day*, *restoring the balance of time*, and *preserving self*. These actions are intended to overcome problems and master changes in order to maintain balance in daily life through constructions that recreate normality and predictability. The third concept, *reconciliation*, was constructed from three categories: *feel normal*, *set to adjust*, and *be positioned*. These categories describe how the participants minimize their experiences of discontinuity by recapturing approaches in order to reconcile with various changes and maintain continuity in daily life. Our findings provide a fresh perspective on continuity that may contribute to the development of significant interventions in continuity of care for persons with CHF. However, continuity requires that healthcare systems support each patient's ability to manage change, reorientation, and adjustment to the new situation in order to make it easier for the patient to create and continue living their daily lives as they desire.

Persons with chronic heart failure (CHF) live with a complex clinical syndrome of symptoms based on their hearts’ lack of supporting circulation in the body (Krum & Abraham, 2009). Living with a failing heart entails an unreliable and inadequate body, affecting daily life, health perception, and well-being (Brannstrom, Ekman, Norberg, Boman, & Strandberg, 2006; Falk, Granger, Swedberg, & Ekman, 2007; Nordgren, Asp, & Fagerberg, 2007; Yu, Lee, Kwong, Thompson, & Woo, 2008). According to Jeon, Kraus, Jowsey, and Glasgow (2010) and Yu et al. (2008), experiences of living with CHF have mainly been studied from a qualitative research perspective and are mostly characterized by various kinds of dysfunctions and limitations with compromised physical and social functions. Living with CHF may also involve an unpredictable daily life due to losses, interruptions, insecurity, and experiences of discontinuity (Burstrom, Brannstrom, Boman, & Strandberg, 2012; Davidson, Dracup, Phillips, Padilla, & Daly, 2007). These experiences are often described from a misery perspective; therefore, it seems important to counteract this viewpoint with a more positive perspective by studying how persons bridge the misery and what contributes to continuity in their lives.

The word *continuity* is explained as “the state or quality of being continuous” in relation to various contexts, according to the *Oxford English Dictionary* (2015). This term can be understood as a flow without interruptions or changes, which provides security in life and can have both positive and negative effects. According to Atchley (1989), continuity is an elusive concept that in one way means to remain the same or be uniform or unchanging. However, this static view of continuity can be difficult to fit into a constantly changing modern life. Continuity can instead be seen as a dynamic process where changes are a part of life; from this viewpoint, adaptive strategies based on past experience are used to deal with changes and create stability in life. Another way to understand a person's multifaceted experiences of continuity in life is from a philosophical point of view. Smith (2010), describes how continuity seems to be a prerequisite for human life, and how it is central to achieving our personalities in relation to social structures. This process occurs by maintaining relationships across time and space, through both daily routines and social relations.

According to Cohler (1982), continuity through the course of one's life is first and foremost a subjective perception; changes are linked to this perception, and it fits in with the individual's personal history. The continuity theory of normal ageing by Atchley (1989) defines continuity as an adaptive strategy that promotes both individual preference and social approval. According to this theory, continuity has both internal and external dimensions. Internal continuity contributes to creating a person's identity (Lieberman & Tobin, 1983) and external continuity contributes to personal growth in relation to other persons and environments over time (Gutmann, 1988). According to Atchley (1989), experiences of both continuity and discontinuity occur within a person's life process due to various changes; such changes can be considered as both negative and positive in relation to continuity.

Continuity is frequently studied in relation to care, usually with a focus on how patients experience continuity with healthcare contacts (Haggerty, Roberge, Freeman, & Beaulieu, 2013; Uijen, Schers, Schellevis, & Van den Bosch, 2012; Waibel, Henao, Aller, Vargas, & Vázquez, 2012). However, studies on life continuity that are related to chronic illnesses such as chronic kidney disease, lung cancer, and stroke describe how the participants experience disruptions in their lives as discontinuity, and how they try to maintain biographical continuity (Becker, 1993; Leveälahti, Tishelman, & Öhlén, 2007; Llewellyn et al., 2014) by using various resources such as pre-illness routines or reconstructed identities (Hinojosa, Hinojosa, Boylstein, Rittman, & Faircloth, 2008). These findings about disruptions in life may be applicable to persons who live with the progressive syndrome of CHF due to the periods of deterioration they experience, which include frequent and long-lasting healthcare contacts or acute hospitalization (Liao et al., 2007; Mejhert et al., 2013). However, to our knowledge there are no studies that describe continuity in daily life from a comprehensive subjective perspective for persons living with CHF. There is a lack of knowledge about how persons with CHF relate to continuity and discontinuity in their daily lives. What is the importance of continuity and what kinds of actions are used to manage discontinuity? The aim of this study is to illuminate whether persons with CHF experience discontinuity in life and, if so, what helps them to preserve and strengthen continuity in their daily lives.

## Method

Grounded theory method (GTM) was used in this qualitative study in order to identify psychosocial processes and actions to maintain continuity in daily life (Charmaz, 2009). An inductive design was chosen in order to deepen the understanding about what is happening in a specific context. The design uses a systematic approach to collect and analyse data; furthermore, it constructs concepts and sets up a formative theory based on the data (Bryant & Charmaz, 2007; Charmaz, 2006; Glaser & Strauss, 1967). The theoretical perspective of social constructionism underlies our method, and we use symbolic interactionism to represent a constructionist perspective, assuming that persons construct their selves, society, and reality through interaction with other persons (Blumer, 1969; Mead, 1934). This perspective contributes depth, power, and relevance to an understanding of how a person with CHF constructs and acts from his or her own reality (Charmaz, 2006, 2009).

### Participants and settings

The participants were purposively recruited from different settings: primary healthcare settings, a specialist clinic at a county hospital, and a local heart and lung association (HLA). By capturing various contexts, different phases of the disease, and different treatments and experiences of living with CHF, we hope to gain as broad a picture as possible. The selection comprised eight men and five women from primary healthcare and from a specialist clinic, added with five women from the HLA. The participants ranged in age from 62 to 88 years (with a mean age of 76 years). All participants had lived with CHF for at least 6 months and some for more than 5 years. The severity of the disease ranged from being almost symptom-free to living with a significant symptom burden. All participants were retired, with 13 cohabiting and 5 living alone. The selection criteria included participants aged ≥20 years, who had been diagnosed with CHF, understood and spoke Swedish, and lived in standard housing. The diagnosis of CHF was confirmed from the participants’ medical records, in accordance with the criteria from European Society of Cardiology (ESC) guidelines (McMurray et al., 2012).

### Data collection

During the period February 2011 to September 2012, data collection was initiated by sending the participants an information letter. After 1 week, they were contacted by telephone regarding the request for participation. Information about the study was provided, and an appointment for the interview was set. Data was collected as audiotaped interviews, and at the end of each interview, all participants gave their permission to be contacted once more if anything in the interview needed clarification. Individual interviews were used to allow participants to verbalize their stories based on their own thoughts, emotions, and actions in order to increase their awareness of their own experiences. Together with the interviewer's experience, the interviews were expected to create a common understanding. Individual interviews lasted for 25–75 min (40 min on average) and were conducted in the participants’ homes or at a primary care setting, according to the participants’ wishes. The individual interviews were the main basis for data collection, although a group interview was also used as a complement to them. The conversation in the group interview between persons sharing a common experience contributed as a supplement and enriched our understanding. The group interview took place in the HLA meeting room and lasted for 100 min.

In both the individual and group interviews, an interview guide consisting of a printed set of questions was used to support data collection and to structure the interviews on continuity and discontinuity in daily life. In order to cover the most important areas of interest, the interviews began with broad-based, open questions with follow-up probes. Prompts such as, “Please tell me what it is like to live with CHF” and “Please tell me how you make your life cohesive, despite illness” were used. Participants were encouraged to talk freely, and there was a flexible use of the interview guide due to the participant's narratives, because some spoke freely, whereas others were more reticent. The participants’ stories about living with CHF, combined with the interviewer's experiences as a nurse in primary healthcare, guaranteed substantial, relevant data. This paper is part of a larger study about continuity in life. Thus far, the collected data has contributed to two manuscripts with different aims, one of which is published (Östman, Jakobsson Ung, & Falk, 2015).

### Data analysis

The data was transcribed verbatim, and the analysis began after each interview. Through the whole process, data collection and coding was carried out simultaneously in order to explore, deepen, and refine questions, a process that contributed to the formation of new questions. A constant comparative method was used in order to systematize and analyze data, compare meanings, and identify similarities and differences (Charmaz, 2006, 2009; Glaser & Strauss, 1967). The constructions of codes, categories, and concepts were carried out continuously in order to be more specific, until no further insights were found (Charmaz, 2006; Morse, 2000). To gain a broad understanding of the whole, the texts were read carefully and then an open coding began. Texts were read line by line and labeled with codes based on phrases or names that were as close to the data as possible. Then, the codes were compared with each other on the basis of similarities and differences. Once all the codes had been identified and were starting to stand out, the next step of focused coding was followed in order to organize and group the data into temporary categories. Step by step, in line with the constant comparative method, the analyses was brought to a more abstract level that generated concepts, confirmed by the properties of the data. During the whole process, memos were used to record reflections, questions, and ideas. This process enabled to build new ideas and identify gaps in the data collection as well as to clarify the relationships between the codes and categories.

### Ethical considerations

Approval of the study was obtained from the regional Ethical Review Board in Gothenburg (Dnr.543-10) and was consistent with the principles outlined in the Declaration of Helsinki (World Medical Association [WMA], 2013). Participants were informed in writing and verbally regarding the aim, procedures, and contact details. For those who wished to participate in the study, written informed consent was obtained before the interviews began. The participants were informed of their right to withdraw at any time, and were told that although the interviews would be recorded and transcribed, no identification data would be used in order to ensure confidentiality. During the interviews, we showed respect for the participants’ conditions and, if unpleasant experiences occurred during or after an interview, we offered participants telephone contact with a counsellor for further follow-up.

## Results

Living with a chronic disease like CHF resulted in disruptions, losses, and setbacks in the participants’ daily lives. Common-sense perceptions of corporeality, temporality, and identity were changed as the disease occupied daily life, leading to *experiences of discontinuity*. To manage these *experiences of discontinuity*, the participants attempted to solve and overcome the problems that occurred in daily life. We identified and defined these actions as *recapturing approaches*, which aimed to recreate corporeality, temporality, and identity in order to fit in with personal preferences and social approval. If the participants’ actions of making their own perceptions compatible with their new daily life situations were carried out successfully, it contributed to reconciliation in relation to corporeality, temporality, and identity, which then helped to reinforce continuity in life.

**Figure 1:**
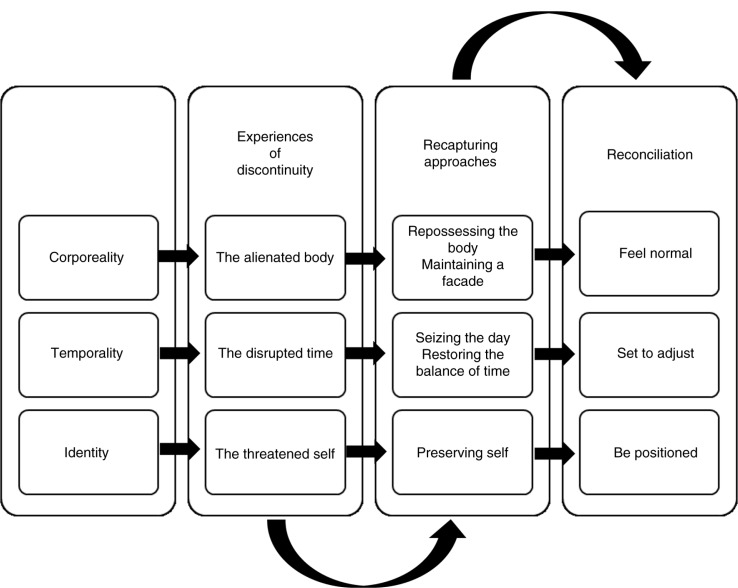
Experiences of discontinuity and creative actions to preserve continuity in daily life.

Because the purpose was to identify processes and actions that help to preserve and strengthen continuity in daily lives, the results are explained based on the concepts: *experiences of discontinuity*, *recapturing approaches*, and *reconciliation* in relation to corporeality, temporality, and identity, to put it in context (Figure 1).

### Experiences of discontinuity

The concept experiences of discontinuity is constructed from the categories of *the alienated body*, *the disrupted time*, and *the threatened self*.

*The alienated body* comprises the participants’ experiences of living with a variety of severe symptoms and an unreliable body that they do not recognize. Physical limitations in relation to corporeality mean that they are constantly thrown back in their own bodies, and that the disease is permanently present. These consequences are described as failing stamina, physical limitations, and a lack of trust in their bodies to function properly; they lead to a sense of being expelled from life. One of the male participants, Bertil, aged 80, described it as follows.

The way things stand when it comes to heart failure means that I don't have the energy to do a huge number of things I would like to do. I get tired quickly and I am terribly sleepy. I always used to wake up in the morning and get up, but now I simply turn over and sleep until 10 am … The heart failure is holding me back. I don't dare to go out and drive long distances, because I get tired and I can't rely on being able to drive home … So I have to limit my activities, like working on the house and garden and so on.

*The disrupted time* refers to temporality and is described by participants as the disease consuming time, and as the natural life cycle becoming discontinuous as it changes from a process to linear time perception with a beginning and an end, where time is lost and never regained. This linear time is characterized by the performance of actions within an allocated, measurable, period of time, which creates discontinuity in connection with the discrepancy between the ability and the inability to realize targets. *The disrupted time* is described using the categories *disruption of daily life* and *end of lifetime*.

*Disruption of daily life* is perceived as daily life being disrupted by the need to set aside time for healthcare visits, medication, and treatment and as the experience of normal, trivial activities making demands and interrupting the daily course of life. According to the participants, this disruption creates discontinuity in relation to temporality and manifests itself as life becomes more difficult to manage. The participants fear having insufficient time, as their perceptions of time during various activities do not match their previous experiences. Every activity takes a longer time and this simultaneously increases change-over times, as more time needs to be allocated to preparations and organization in connection with activities. This disruption creates conflict that generates stress or apathy. Sarah, aged 65, described it as follows.

I feel that I have a certain limited strength and how should I use it? If I should go here and clean up then I cannot go to the doctor, dentist or any other activities, yet it is not certain that I can do it either… Everything takes a longer time, it is difficult to manage daily activities like putting on clothes, showering and washing your hair. Showering takes an hour at least, dressing can take a half hour and putting on shoes can take fifteen minutes. So you may start getting dressed three hours before you go somewhere simply and easy, and then three hours have disappeared.

*End of lifetime*, on the other hand, relates to existential disruptions and discontinuity in the living process that are related to being aware of one's own death and limited future. In relation to temporality, it leads to consequences such as doubts about the future and death, and is described as living with restricted time and in a state of uncertainty. Participants express this as painful, as they are unable to find a solution to their own uncertainty about the future. The future feels inaccessible as a result of all the obstacles; so participants stop planning their daily lives and put their future plans on the shelf. The experience of time as wasted and pointless is described as having “dead” time, as circumstances outside the participants’ control produce passivity. Bertil, 80, expressed it as follows.

The worst thing is that nothing gets done, it's hard to get going, to get something out of your hands, really hard. I do not dare to do things and I can't stand it, it's just forced. It has changed my way of living and my view of life. One perceives that it is not time yet. I have more to do before I can give in.

*The threatened self* describes how changes in position in the social context and within the family create a loss of self in relation to identity. Living with CHF results in the consequence of “being someone who is ill,” thereby threatening the person's self-image in relation to him/herself and others. The participants also describe increased loneliness as their social intercourse is reduced because they no longer have the energy to socialize as they did in the past. At the same time, friends and acquaintances withdraw and are less available when illness arises. Changes take place in family roles as the participants become increasingly dependent on their family members to manage their daily lives, and a lack of understanding on the part of relatives leads to conflict as the participants do not receive the support they desire. The experience of not being needed and becoming a burden to the persons around them is perceived as belittling, and has a negative impact on their identities. This experience was described by one of the male participants, Olof, aged 88.

Being someone who is sick, it is quite pointless. Because I am very much a burden for both the family and society. I need help, and for a person like me, who is used to always taking care of himself in private life, family life and work, then it's very hard.

### Recapturing approaches

The concept recapturing approaches describes different processes that create continuity, such as *repossessing the body, maintaining a façade, seizing the day, restoring the balance of time*, and *preserving self*.

*Repossessing the body* deals with the way participants attempt to return their corporeality to a comprehensible, recognizable, entity in relation to their physical conditions and functional capacity. They fight to maintain the sense of a body that functions by comparing themselves with other persons around them who are healthier, more ill, older, or younger than themselves. Olof, 88, one of the male participants, described it like this:

You can compare yourself with a neighbour, for example. He runs like a deer here in the forest and he has no problem with getting out of breath or anything. However, when I was about the same age, I was in much the same shape and he could also develop the same problems as me. I also have acquaintances and old friends who were 20 years younger than me and they aren't even alive.

Another approach involves overcoming physical problems by exchanging similar perceptions and experiences with other persons living with CHF in order to recognize themselves in others and realize that they are no different. One of the women, Rut, 72, described how advice from other persons in the same situation had supported her in managing her daily life.

Unfortunately, I became irritated really easily, but I attended a course with seven other heart failure patients. Your mood, being easily irritated and so on, clearly has something to do with the heart, and we all felt the same way … We complain when it's windy, because it's really difficult, and someone said that we should buy one of those masks bank robbers have that cover your mouth, but you can also use a scarf, as it isn't easy when it's windy. It really worked!

When the participants fail to match their own expectations with a real-life situation in relation to their corporeality, they attempt to recreate the way things used to be. This is done by living as close to a previous daily life situation as possible by developing activities and skills that still function. To quote Arne, 79, one of the male participants:

You have to learn to listen to your body's signals and do things at a tempo you can manage. I also try to make my daily life easier. I have done and I am still doing this, I'm developing the whole time.

*Maintaining a façade* describes the way participants create actions to balance and minimize their physical limitations in order to maintain a kind of normal corporeality. This contributes to “putting a brave face on things” by expressing gratitude for and satisfaction with their situation, as it could be worse. Bo, 69, describes it as follows:

I think about persons with cancer. I go to see a friend who has prostate cancer, so there are plenty of things I think are far worse than what I have. I feel that I should be grateful that I only have heart failure.

They also try to give the impression of being strong by creating a façade that matches the healthy person they used to be, even if it means making up imaginative stories about their physical abilities. Olof, 88, illustrated this in the following quote.

As I see it, you shouldn't sit still. You have to try to get moving, as quickly as you possibly can … and I have always made an effort to and enjoyed managing. At physiotherapy, you remember what it was like to be young, that you have to do your best. So, when the physiotherapist puts 20 kilograms on a machine, I add another 20 to show just how good I am and that isn't good, because it burns and I can have pain for several days. My whole body hurts.

At the same time, participants construct rational explanations about their corporeality to make their illness comprehensible by attributing the causes and effects to physical limitations. These limitations are usually their age or physical shortcomings caused by problems with joints or muscles. Henry, 78, explained it like this:

It's my ability to move. That's due to my knees. I can't cut the grass, so my wife does it, but I cook instead. Difficulty moving means I can't do the things I used to at home, if you like, and it's due in equal part to my knees and my heart.

*Seizing the day* relates to temporality by the way participants attempt to manage disruptions in time and in their actual life processes. They try to live here and now, taking each day as it comes and minimizing their demands, by focusing on small details. They attempt to make the most of the time they have left by filling their daily lives with the things they enjoy doing most, even if they have to adapt their leisure interests by choosing quieter activities or finding new and inspiring interests on which to focus. This is what Kurt, 73, one of the male participants, had to say:

I dance as much now as I have ever done. I shall never stop, as long as my legs are able to support me, I love it! I have never had as much fun in my life as I have now. I meet other persons and I realize that I'm a good dancer. This is proved by the fact that ladies come and ask me to dance. Goodness me, it's such fun! When I was younger and had more energy, I enjoyed old-time dancing, but I can't manage that anymore because my energy runs out. So I do the foxtrot instead.

*Restoring the balance of time* is about maintaining temporality by creating stability in life through setting targets for the day and achieving a sense of meaningfulness, even if the targets differ from those prior to becoming ill. This stability may mean continuing to plan your day but doing so on a more short-term basis, by not planning as though time is infinite. One of the women in the group interview, Anna, 75, put it like this:

I take a week at a time and only plan for next week. I always say “We shall have to see”, if one of my grandchildren asks if I am coming to a party. One of my grandchildren is getting married and she asked me if I was going to make her wedding cake. So I told her it would depend.

Temporality is a question of constantly balancing between being able and being unable to perform planned activities. The participants attempt to adapt their plans to match their abilities in order to avoid being disappointed. They do this by dividing their activities during the day, refraining from or adapting physical activities, only doing one thing at a time, or obtaining assistance from other persons. Lisa, 64, one of the other women in the group interview, gave the following description.

It's incredibly important when you are like this and get one ailment after another. You have to think about doing things at your own pace, in your own environment, what you really want or do nothing at all … In the past, I used to go to the hairdresser and then do errands in the town afterwards, but I can't do that now. I can only manage the hairdresser and so I have to forget about the other things, postpone it and do it some other time.

*Preserving self* relates to identity and describes the way participants attempt to preserve their self-image to themselves and to others by actively safeguarding their profiles. They do this in different ways that are both avoidance-based and strengthening. One way is to question their illness and to maintain their identity as healthy, because the role of someone who is ill doesn't match their own perception of themselves. Another way is described as lending a helping hand to others around them to feel needed, while simultaneously trying to strengthen their positive characteristics in order to be seen as happy, humorous, talkative, and reliable. They also focus on maintaining valuable skills such as still being a popular photographer, cook, parent, or friend. Lennart, 76, put it like this:

The only real change that has taken place since I became ill, let's say, if I am ill, is that I cook all the food and I didn't do that before. I have never done it, but there are food programmes on TV the whole time and I love cookery books. I buy a large number of cookery books and we have a lot of them. Sometimes I actually read them and I think it's fun, I have to admit.

The participants describe how they endeavour to maintain identity by being part of their shared world, regardless of their illness. They express how their relatives provide support and act as a “glue” to connect them to their social context during changes in the rhythm of their lives. This experience is illustrated by the following quote from Henry, 78.

I usually always ask my wife what we are doing this week. We are going to pick up the grandchildren today, and then we are going to take them home and fix the food, because our daughter works. We help out a great deal and we have to book these activities first. Afterwards, we can decide if we are going to do something else on one of the days. I sometimes find it difficult having small children at home, they rush backwards and forwards and it's disruptive, but I don't want to stop doing it.

The participants attempt to maintain contact with other persons in order to preserve identity. As a result, they take part in different activities for as long as they can manage to do so. Another way of maintaining contact with others is to find new opportunities using social media. Here is what Lennart, 76, had to say:

My friend Janne and I, we talk on the phone perhaps twice a month. Janne and I have had the same interests all our life. We have hunted together, fished together, taken part in sports together and so on. You need to take care of your friends, even if you don't meet them that often.

### Reconciliation

The concept reconciliation is made up of the categories *feel normal*, *set to adjust*, and *be positioned*. It describes how participants attempt to minimize discontinuity by reconciling themselves with various changes in their lifestyles.

*Feel normal* relates to how participants attempt to reconcile themselves with their corporeality in response to experiences of “losing” their bodies. They work to achieve normality in relation to different physical comparisons and attempt to reconcile themselves with the prevailing circumstances by focusing on the functions that have not been affected or changed. At the same time, realizing that it is impossible to cure the heart, they create a sense of security in their daily lives that is based on an attitude of “it is what it is”. In this way, they create a sense of physical equilibrium and normality, which promotes the normalization process. Vera, 82, who was one of the women in the group interview, described it as follows.

It's not possible to repair me, the heart is impossible to repair… However, I'm quite mobile even though I use a walker. But it's not possible to do any miracles with me, and I don't demand it either.

*Set to adjust* describes temporality as an aspect of daily life that is perceived as both cyclical and linear. This temporality then contributes to either continuity or discontinuity, depending on the participants’ attitudes to their own thoughts and activities. Realizing that life is limited makes it easier for participants to understand the discrepancy between hope and reality in terms of time; and achieving synchronicity contributes to integrating their illness with their ongoing lives. Integration is first and foremost about being aware of the consequences of their illness in their daily lives and thinking rationally about their illness and their functional impairment. Understanding the need to set aside time for rest in connection with activities and finding a balance between functionality and resources in relation to expectations and requirements supports them in creating less problematic transitions in their daily lives. This is what one of the women, Lisa, 64, in the group interview said:

I may just say that you simply have to readjust, and live in a completely different way, you have to constantly keep in mind that you can't do too much and you always have to adapt yourself in relation to time. You simply have to readjust and rethink in life.

*Be positioned* relates to identity, and describes how the participants interact with other persons in the attempt to preserve their previous identity; that is, the way they perceived themselves and the way others perceived them before they developed their illness. Participants attempt to reconcile themselves with the change in their identity by preserving their personality, roles, social activities, and relationships, regardless of the impact of their illness. When family members take over tasks they previously performed, relationships change; this produces both continuity and discontinuity, depending on how the change influences participants’ self-image. Continuing to be part of their social context makes the participants socialize and interact with family, friends, and other significant persons and contributes to maintaining continuity in their life story. When their image matches with what they want to be true, it promotes a sense of maintaining their social position in the world. Reconciling with their current life can create happiness and preserve their self-image, in spite of the magnitude of the changes that occur. *Be positioned* is described in the following way by Lisa, 64, in the group interview.

I am a member of the health team. We take blood pressure in different places, so I meet a huge number of other persons who are active in associations, as well as who come to visit me. It's really enjoyable and a lot of fun, you feel much stronger when you have been there. You can say, “God, I managed that, first and foremost, I managed”. First of all, you aren't sitting at home, feeling sorry for yourself because you can't manage anything. You are out among others, you're yourself in a different way … It was such a setback when I became ill, after having had a job for the whole of my life. But then I got a new kick-start in my life, that's what's enjoyable. When I worked, the demands were different. Now we meet and have fun together and then I go home late in the afternoon and I have done something with my day. I get home much earlier and yet I have still had this stimulation.

## Discussion

The main finding in our study was how various constructions and actions made participants continue living and acting in a familiar way. This familiarity gave participants a sense of security and predictability both to themselves, to others, and to their surroundings which strengthened their experience of continuity in their daily lives despite the various alterations caused by CHF. It seems to be of great importance to exist and interact in well known environments in accordance with others in order to perceive continuity when living with CHF which is in line with the continuity theory of normal ageing by Atchley (1989). Perhaps the most striking insight is that participants continued to strive to create solutions no matter what happened, and never gave up on attempting to maintain their continuity in life. The concept illustrates participants’ *experiences of discontinuity* due to CHF and its consequences to their corporeality, temporality, and identity; however, it also illuminates how they use *recapturing approaches* and seek *reconciliation* in order to preserve continuity in their daily lives.

In the interviews, participants expressed that “life is generally a bit more difficult,” due to a range of symptoms and functional impairments that they perceived as disruptions and distressing losses. These losses and limitations in relation to the alienated body, disrupted time, and threatened self, revealed a seemingly hopeless situation of fear and insecurity in daily life. This perception of hopelessness is partly consistent with previous studies about the impact of CHF on daily life (Jeon et al., 2010; Welstand, Carson, & Rutherford, 2009; Yu et al., 2008) and the experience of “loosing normal life” seems to be common when living with chronic illness (Larsson & Grassman, 2012; Najafi Ghezeljeh, Yadavar Nikravesh, & Emami, 2014). This is in line with our findings, though we can add that it contributes to *experiences of discontinuity* in relation to corporeality, temporality, and identity.

We found that *experiences of discontinuity* in relation to corporeality, temporality, and identity can be stressful experiences that contribute to both practical and existential issues in life. Whereas the contrary, continuity seems to be a prerequisite for human life, and is central to achieving our personalities in relation to social structures, according to Smith (2010). Thus, we can argue that living with changes, disruptions, and losses due to CHF has negative consequences for the continuity of life, according to the participants’ narratives. Nevertheless, *experiences of discontinuity* made the participants to structure and organize their lives to mitigate disruptions by creating *recapturing approaches*, in order to maintain continuity in their life situations. These findings may be linked to Antonovsky's (1987) sense of coherence, given how the participants used various actions in order to achieve comprehensibility, manageability, and meaningfulness in daily life.

The findings of recapturing approaches when living with CHF include various processes such as: repossessing the body, maintaining a façade, seizing the day, restoring the balance of time, and preserving self. These actions intended to create continuity in life and could be both avoidance or confrontational, depending on the situation. This shows similarities with a literature review (Ambrosio et al., 2015) which describes the concept of living with chronic illness, even if it is not based on a continuity perspective. According to Ambrosio et al. (2015), living with chronic illness involves various processes from disavowal to acceptance with intention to manage daily life which is reinforced by other studies of living with chronic illness (Larsson & Grassman, 2012; Najafi Ghezeljeh et al., 2014) and CHF (Welstand et al., 2009). However, our findings add how these actions contribute to *reconciliation* from a continuity perspective which supports continuity in life when living with CHF.

Our findings showed that participants sometimes tried to keep their diagnosis of CHF a secret by hiding behind a façade, and that they tried to reconcile with their unpredictable bodies by constructing explanatory models to comprehend their illness. These façades and explanations acted as a counterforce to participants’ *experiences of discontinuity*, and their narratives were sometimes astounding. However, these narratives could also be a way of avoiding dealing with their unpredictable bodies, due to their fear of being overcome by CHF. These findings are highly coherent with previous studies about chronic illness by (Charmaz, 1990, 1991, 2002), even though those findings were not about CHF. Another study by Helvik, Iversen, Steiring, and Hallberg (2011) describes how elderly persons with somatic health problems calibrate and adjust their expectations in life in order to maintain control and balance over reduced energy and health. These findings seem to be comparable to our participants’ *recapturing approaches*. However, perhaps it is not the diagnosis that determines the actions, but rather the experiences of discontinuity caused by bodily limitations in daily life.

The participants expressed that time was running out, a perception that intruded on daily life both practically and existentially. These disruptions seemed to make time more precious to participants than before. The experiences participants reported of their sense of time changing are in line with other studies about experiences of living with chronic illness (Aujoulat, Luminet, & Deccache, 2007; Charmaz, 1991, 2003; Hansen et al., 2012). However, we focused on how the participants’ constructed actions related to disrupted time by seizing the day and restoring the balance of time. Even though everything changes in life, participants strove to take control by organizing their time based on their conditions in order to maintain continuity in daily life.

Living with CHF affects participants’ identities especially when friends and acquaintances pull away. The unwanted decrease in social interaction contributes to questions about why other persons withdraw, and leaves participants with a sense of loneliness that is similar to experiences described in previous studies (Nordgren et al., 2007; Yu et al., 2008). These alterations lead to disconnectedness from everyday social networks and support systems, thus creating discontinuity in life. In contrast, however, connectedness to a family, social context, and known environment helps to put the disease into the background and come to *reconciliation*. This is in line with Bury's (1982) findings about how biographical disruptions in daily life result in participants mobilizing resources and maintaining social relationships in order to decrease their social isolation and recreate a new way of living, which is supported by other studies (Larsson & Grassman, 2012; Najafi Ghezeljeh et al., 2014; Welstand et al., 2009). These similarities confirm our results and reinforce the importance of supporting persons with CHF in their actions to maintain continuity in their social relationships.

Our findings show how persons with CHF struggle to achieve a normal life—a process that is not entirely straightforward. It requires strength, power, and ingenuity to maintain continuity in daily life when living with CHF. We found that daily life with CHF becomes manageable as participants reach understanding and confidence through *reconciliation* with their corporeality, temporality, and identity. This includes the categories: feel normal, set to adjust, and be positioned. It seems to be possible to maintain continuity in daily life as long as participants make changes that align with their personal preferences and social requirements, because changes do not necessarily imply discontinuity. According to the continuity theory of normal ageing by Atchley (1989), continuity of self and continuity of identity are not difficult to achieve for the elderly, because older persons accept themselves more readily as not being how they might like to be. However, Atchley's theory focuses on normal ageing and does not take chronic illness, with its gradual or acute deterioration, into account.

Our results show that continuity is not only about maintaining continuity of self; it also involves conforming to what other persons expect. Therefore, continuity seems to be more difficult to achieve when living with CHF, with its losses, disruptions, and insecurity, than in normal ageing. This can be compared to a study by Larsson and Grassman (2012) who argue that the only thing that stands for continuity when living with chronic illness, are more or less repeated disruptions. However, this is not consistent with our findings, because we can show that persons with CHF create *recapturing approaches* as far as possible to come to *reconciliation* and achieve continuity in daily life despite the fact that they are living with CHF. Therefore, we argue that our results contribute new knowledge to the field of continuity experiences.

### Methodological considerations

Our results are based on participants’ experiences of living with CHF. Using GTM, we explored strategies, actions, and psychosocial processes that expressed a sense of continuity in daily life. According to Charmaz (2006), using GTM enables the collection of detailed and contextual data from participants’ unique perspectives. Having continuity as a sensitizing concept enabled us to answer our research question about the meaning of continuity and the process of preserving continuity in daily life.

The concepts discussed in this paper were constructed from qualitative interviews based on participants’ explanations of how they dealt with different situations based on their reality, dreams, and fantasies. Our intention was to give voice to the participants’ experiences; it was not our mission to analyse what was true or false in their narratives. However, we are aware that some of the actions mentioned by participants in this study are hardly feasible due to imbalances in resource supply and demand.

We have attempted to attain trustworthiness by describing our method and by analysing the concepts in detail using quotes from the interviews. We have achieved credibility by carefully following the steps in the GTM. This study was conducted by gathering rich data, continually comparing codes and categories, performing repeated critical reviews of the analysis, and cross-checking between the authors; it also involved an awareness of the impact of the participants’ interactions and the researchers’ reflexivity. New insights into continuity in daily life when living with CHF have assisted us to reach originality in our study. With regards to resonance and usefulness, our findings increase knowledge and provide new and relevant information on the context of continuity in daily life when living with CHF, and our findings may be applicable to other chronic diseases.

The interviews took place at a single point in time, and we did not follow the participants over time; this can be seen as a limitation of the study, because we are unable to say whether participants experienced changes in their perception of continuity over time and as their conditions deteriorated. On the other hand, we have tried to capture persons in various stages of CHF, which could compensate for this limitation. Another limitation might be the number of interviews. However, using individual interviews with persons from different healthcare contexts along with a group interview supported us in obtaining a broad picture of the studied area with thick, rich, and useful data. The high mean age of the participants (76 years) and the comorbidity may also be a limitation. However, the mean age in this study is representative for the population that lives with CHF, as this disease affects elderly persons more widely than younger ones, and elderly persons often live with more than one chronic disease (i.e., diabetes, vascular disease, and lung disease), according to other studies (Jhund et al., 2009; McMurray et al., 2012; Yancy et al., 2013).

## Conclusion and implication

When one's life changes due to CHF, and when what was previously taken for granted becomes unreliable and uncertain, life seems to be more difficult to live. According to our findings, living life with CHF involves *experiences of discontinuity* in relation to corporeality, temporality, and identity in many aspects. However, such experiences make participants strive for continuity by creating *recapturing approaches* to prevent discontinuity. This is an ongoing process that attempts to create *reconciliation* with the new situations in participants’ daily lives. Thus, our findings generate a deeper understanding about continuity in daily life and indicate that it should be a therapeutic reality in healthcare for caregivers to focus on a person's capacity to create continuity in his or her daily life according to that person's own assets. Such a reality requires healthcare providers to see and support each patient's ability to manage changes, reorientations, and adjustments to his or her new situation. This study provides an additional perspective on continuity that may contribute to developing significant interventions in continuity of care for persons with CHF.
